# Conservative Management of a Second-Trimester Cervical Ectopic Pregnancy

**DOI:** 10.31486/toj.20.0034

**Published:** 2020

**Authors:** Katelyn F. Handley, Lauren M. Bergeron, Joseph R. Biggio

**Affiliations:** ^1^ Department of Obstetrics and Gynecology, Ochsner Clinic Foundation, New Orleans, LA; ^2^ The University of Queensland Faculty of Medicine, Ochsner Clinical School, New Orleans, LA

**Keywords:** *Amniocentesis*, *dilatation and curettage*, *fertility preservation*, *pregnancy–ectopic*, *ultrasonography*, *uterine artery embolization*

## Abstract

**Background:** Cervical ectopic pregnancy is a rare condition, historically treated by hysterectomy.

**Case Report:** A 33-year-old female at 13 weeks 3 days’ gestation was diagnosed with a cervical ectopic pregnancy. She underwent a uterine artery embolization, fetal intrathoracic potassium chloride injection, amniocentesis, and ultrasound-guided suction dilation and curettage with the use of intracervical vasopressin, flowable gelatin with thrombin, and cervical cerclage.

**Conclusion:** Advanced cervical ectopic pregnancy can be successfully managed in a conservative fashion in a patient who strongly desires future fertility.

## INTRODUCTION

Cervical ectopic pregnancy is a rare occurrence, arising in 1:1,000-18,000 pregnancies.^1-3^ Known risk factors include history of dilation and curettage (D&C)—preceding 60% to 70% of cases—and advanced reproductive technology.^4-8^ Ninety-one percent of women with cervical ectopic pregnancy present with vaginal bleeding that is typically painless, thought to be secondary to relatively decreased pain receptors in the cervix. However, 29% present with massive hemorrhage.^7^ The differential diagnosis typically consists of spontaneous or missed abortion and can sometimes be confused with cervical cancer on physical examination with a markedly vascular, friable, barrel-shaped cervix with a partially opened external os.^1,2^

We present a case of cervical ectopic pregnancy that was successfully managed in a conservative fashion in a patient who strongly desired fertility.

## CASE REPORT

**Figure 1.**
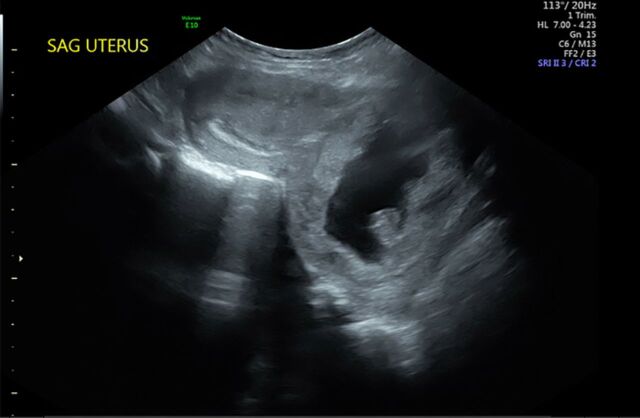
**Sagittal (SAG) ultrasound of cervical ectopic pregnancy at 13 weeks 3 days’ gestation, demonstrating an hourglass-shaped uterus with a ballooned endocervical canal, gestational tissue at the level of the cervix, and empty uterine cavity.**

**Figure 2.**
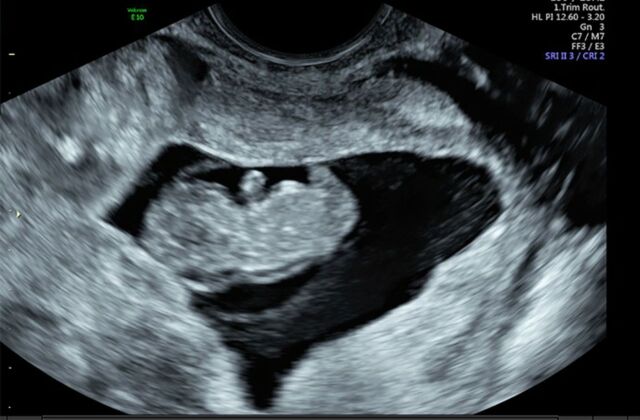
**Ultrasound demonstrating fetal pole consistent with 13 weeks’ gestation.**

**Figure 3.**
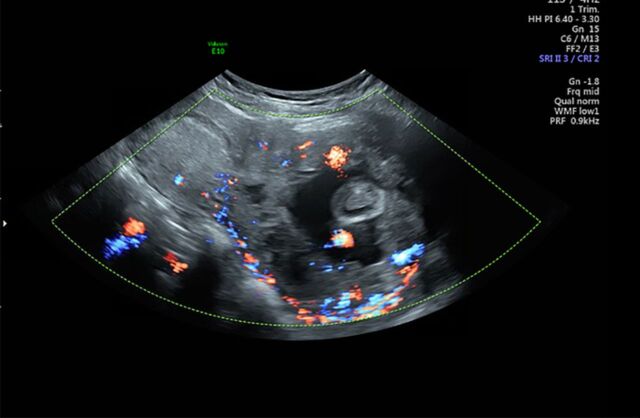
**Ultrasound with Doppler flow demonstrating the vascularity surrounding the cervical ectopic pregnancy.**

**Figure 4.**
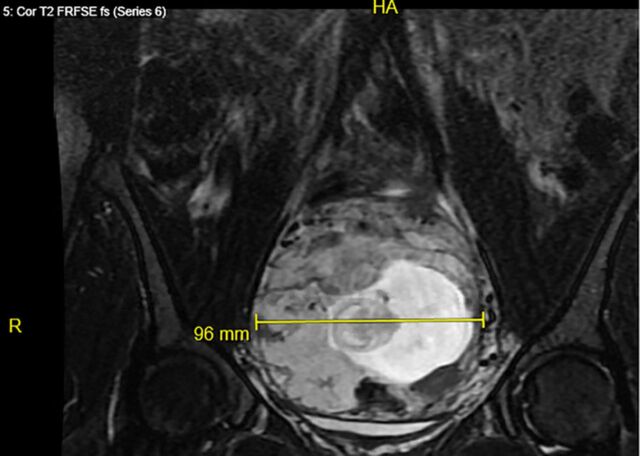
**Coronal pelvic magnetic resonance imaging demonstrating the cervical ectopic pregnancy with measurement of the gestational tissue mass (96 mm).**

**Figure 5.**
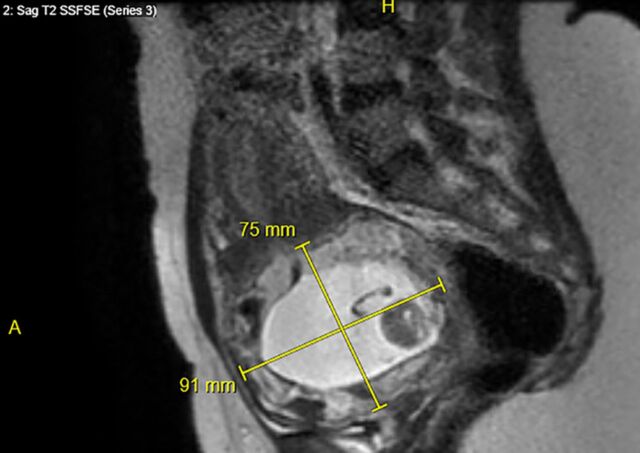
**Sagittal pelvic magnetic resonance imaging demonstrating the cervical ectopic pregnancy with measurement of the gestational tissue mass (91 mm × 75 mm).**

A 33-year-old female, gravida 2 para 1 at 10 weeks 2 days’ gestation with a history of a low transverse cesarean delivery, presented as a referral for threatened abortion with a low-lying gestational sac. The patient reported vaginal bleeding since 5 weeks’ gestation. Ultrasound confirmed a low-lying gestational sac with a 4.1-mm cervix and placental hematoma. On repeat ultrasound at 13 weeks 3 days’ gestation, the differential diagnosis included cervical ectopic pregnancy, impending miscarriage, cesarean scar ectopic pregnancy, and abdominal ectopic pregnancy (Figures 1, 2, and 3). Fetal cardiac activity was present. The patient denied pain and current vaginal bleeding. Magnetic resonance imaging demonstrated that the pregnancy was located in the cervix, 6 mm from the external cervical os. The gestational tissue measured 96 × 91 × 75 mm within the 111 × 99 × 106 mm cervix (Figures 4 and 5). Beta human chorionic gonadotropin (hCG) was 67,947 mIU/mL.

A multidisciplinary team that included maternal-fetal medicine, gynecologic oncology, and interventional radiology reviewed the case and recommended uterine artery embolization (UAE) followed by hysterectomy. However, the patient strongly desired future fertility with uterine preservation.

At 13 weeks 5 days’ gestation, the patient had self-limited vaginal bleeding and was transfused 2 units of packed red blood cells. The following day, UAE with collateral embolization was performed with Gelfoam until each artery was embolized to minimal or stagnant flow. The next morning, at 14 weeks 0 days’ gestation, ultrasound demonstrated the presence of cardiac activity. Ultrasound-guided fetal intrathoracic potassium chloride (KCl) injection was performed with fetal asystole noted. Amniocentesis removed approximately 25 mL of amber-colored fluid, decompressing the gestational sac to decrease the size of the cervical mass and reduce the risk of bleeding.

**Figure 6.**
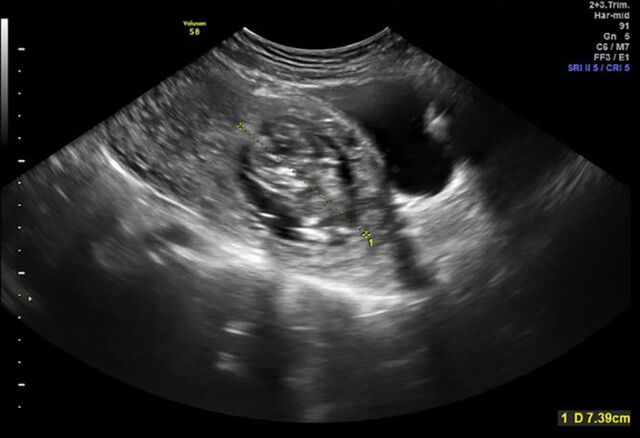
**Ultrasound postamniocentesis at 14 weeks 1 day of gestation demonstrating interval decrease in the size of the cervical mass.**

Follow-up ultrasound 1 day later demonstrated decreased blood flow compared to prior ultrasound. The cervical mass appeared smaller, measuring approximately 95 × 74 × 68 mm (Figure 6). The patient was taken to the operating room for ultrasound-guided suction D&C after counseling regarding the potential need for hysterectomy. Prior to dilation, 20 U vasopressin diluted in 20 mL normal saline was injected into the cervical stroma and at the 3-o’clock and 9-o’clock positions of the cervicovaginal junction. Ultrasound-guided D&C was performed, with an estimated blood loss of 600 mL. Because of the lateral extent of the mass and the attenuation of the cervical tissue, a small amount of placental tissue was left in situ in the right lateral cervix secondary to concern for potential perforation into the uterine vessels with continued curettage. Flowable gelatin with thrombin (Surgiflo, Johnson & Johnson Medical Devices Companies) was injected into the endocervical canal, and a 0 chromic purse-string suture was placed just below the cervicovaginal reflection for hemostasis of the vascular bed, in a technique similar to a McDonald cerclage.

The patient received 2 units of packed red blood cells postoperatively. She received intravenous ampicillin (2 g every 6 hours), gentamicin (5 mg/kg daily), and clindamycin (900 mg every 8 hours) for 72 hours postoperatively and was discharged on postoperative day 5 with instructions to complete 14 days of oral doxycycline (100 mg twice daily). When the patient was seen 4 days after discharge, beta hCG had decreased to 2,743 mIU/mL, and transvaginal ultrasound demonstrated a persistent 80 mm cervical defect.

The patient was seen every 2 to 4 weeks in clinic, and by 4 months postoperatively, the cervical defect had decreased to <20 mm. Menses had resumed, and she was asymptomatic. She was counseled to continue effective contraception for at least an additional 6 months prior to trying to conceive. She was also counseled regarding cervical length surveillance and potential cerclage in a subsequent pregnancy.

## DISCUSSION

The classic and routine treatment for cervical ectopic pregnancy is hysterectomy. In 1968, Mortimer and Aiken stated, “gestations in excess of eight weeks are best treated by abdominal hysterectomy,”^1,9^ and in 2013, Singh observed that 100% of documented cervical pregnancies beyond 12 weeks’ gestation ultimately required hysterectomy.^10^ However, these indiscriminate boundaries have been pushed to accommodate patients’ desires for future fertility.^11^ Approaches to the conservative management of cervical ectopic pregnancy include laparoscopic uterine artery ligation, UAE, methotrexate with or without KCl, curettage and tamponade with or without preoperative cerclage placement, and double-balloon decompression.^12^

Reduction of blood flow to the cervical ectopic pregnancy can be performed via laparoscopic uterine artery ligation or UAE performed by interventional radiology.^13,14^ UAE was our first approach after consultation with interventional radiologists, and a UAE was performed with additional embolization of the collateral branches feeding the cervix and cervical ectopic pregnancy.

Methotrexate is used in the treatment of cervical ectopic pregnancies, particularly at early gestational ages. Similar to relative contraindications more commonly seen with tubal or ovarian ectopic pregnancies, the failure rate is significantly increased in patients with a gestational age ≥9 weeks, beta hCG ≥10,000 mIU/mL, crown-rump length >10 mm, or cardiac activity.^4,15^ Methotrexate can be used alone or with KCl. At a gestational age <12 weeks, methotrexate with KCl and concomitant surgical procedures (as indicated in 34% of cases) result in resolution of cervical ectopic pregnancy and uterine preservation in 91% of cases.^2,4,16^ We did not use methotrexate because the patient met every risk factor for increased failure. When fetal cardiac activity persisted despite the use of UAE, we used an intrathoracic injection of KCl followed by amniocentesis to decrease the volume of the cervical mass, which would be important if the patient required a hysterectomy.

Cervical curettage can be used to evacuate a cervical ectopic pregnancy. Ushakov et al reported reliable hemostasis in 93.8% of cases using cervical curettage in first-trimester cervical pregnancies when preoperative cervical preparation (UAE, ligation of the cervical branches of the uterine arteries, intracervical vasopressin, or a Shirodkar-type cerclage) was used.^7^ Tamponade with an intracervical Foley balloon often follows. Some cases report gradual decompression of the Foley balloon for 24 to 48 hours in a fashion similar to a Bakri balloon.^4,7,17^ Ushakov et al described a 92% success rate for tamponade with a Foley balloon compared to a 61% success rate for tamponade with packing alone. Intracervical vasopressin injection and/or ligation of the cervical branches of the uterine artery with sutures placed at the 3-o’clock and 9-o’clock positions can be performed preoperatively to decrease blood flow.^7^ Prior to D&C, we knew that if we needed tamponade, a Foley balloon would not be large enough. Therefore, we had multiple Foley balloons available, as well as a Cook Cervical Ripening Balloon, which holds 80 mL of fluid, to provide adequate tamponade if indicated.

A cerclage placed at the internal os to compress feeding vessels prior to D&C has been described.^4,17^ We opted to use a similar technique to compress the cervix post D&C to maintain hemostasis by placing a purse-string suture at the upper portion of the cervix.

## CONCLUSION

Using a combination of new and previously reported techniques with minor modifications, we successfully treated this second-trimester cervical ectopic pregnancy and maintained the patient's fertility with uterine preservation.

## Notes

This article meets the Accreditation Council for Graduate Medical Education and the American Board of Medical Specialties Maintenance of Certification competencies for Patient Care and Medical Knowledge.
